# Sex-related differences in conduit strategy and early outcomes after sternum-sparing on-pump multivessel CABG via left anterior thoracotomy

**DOI:** 10.3389/fcvm.2026.1829201

**Published:** 2026-05-11

**Authors:** Volodymyr Demianenko, Hilmar Dörge, Marius Grossmann, Ahmed Belmenai, Christian Sellin

**Affiliations:** Department of Cardiothoracic Surgery, Heart-Thorax Center, Klinikum Fulda, University Medicine Marburg, Campus Fulda, Germany

**Keywords:** cardiopulmonary bypass, coronary artery bypass grafting, minimally invasive cardiac surgery, sex differences, TCRAT

## Abstract

**Background:**

Sex-related disparities after conventional coronary artery bypass grafting (CABG) have been consistently reported, including differences in baseline risk profile, conduit strategy, and early adverse events. However, data on outcomes after sternum-sparing minimally invasive multivessel CABG remain limited. This study aimed to evaluate sex-related differences in baseline characteristics, operative strategy, and in-hospital outcomes in patients undergoing minimally invasive multivessel CABG using total coronary revascularization via left anterior thoracotomy (TCRAT).

**Methods:**

From November 2019 to December 2025, CABG via left anterior minithoracotomy with cardioplegic arrest was the preferred surgical approach in our institution. The analysis included 807 men and 138 women undergoing non-emergency TCRAT. Baseline characteristics, grafting strategy, procedural times, and in-hospital outcomes, including mortality, stroke, myocardial infarction, repeat revascularization, and MACCE, were compared between sexes.

**Results:**

Women were older (69.2 ± 8.9 vs. 66.7 ± 9.4 years, *p* = 0.004) and had a higher EuroSCORE II (3.85 ± 4.70 vs. 2.71 ± 2.51, *p* = 0.006). The extent of coronary artery disease was similar between sexes, with no significant difference in the distribution of 3-vessel and 2-vessel disease. Diabetes mellitus was more prevalent in women (44.2% vs. 34.0%, *p* = 0.026). Total arterial grafting (35.8% vs. 21.7%, *p* < 0.001) and radial artery use (62.2% vs. 35.5%, *p* < 0.001) were more frequent in men, who also received more distal anastomoses (3.17 ± 0.84 vs. 2.92 ± 0.90, *p* = 0.002). Operative, cardiopulmonary bypass, and cross-clamp times were also longer in men. In-hospital mortality (1.12% vs. 0.72%, *p* = 0.676), stroke (0.74% vs. 1.45%, *p* = 0.404), and MACCE (1.87% vs. 3.62%, *p* = 0.293) did not differ significantly between sexes.

**Conclusion:**

Sternum-sparing multivessel CABG via left anterior thoracotomy was associated with similar early postoperative outcomes in men and women despite differences in baseline characteristics and conduit selection. Given the observational design and low event rates, these findings should be interpreted with appropriate caution.

## Introduction

Coronary artery bypass grafting (CABG) remains a cornerstone of myocardial revascularization for patients with complex coronary artery disease, particularly in those with left main disease or high-complexity multivessel disease, where contemporary guideline recommendations continue to favor CABG to improve survival. Despite major advances in percutaneous coronary intervention, surgical revascularization retains unique strengths related to durability and the ability to achieve complete anatomical revascularization across multiple territories, especially in anatomically diffuse disease (1–3). Accordingly, surgical programs increasingly focus on optimizing perioperative safety while preserving the long-term advantages of CABG.

Over the past decade, a consistent body of evidence has highlighted clinically relevant sex-related differences in CABG, in line with recommendations to account for sex and gender equity in biomedical research (23). Women typically present for surgery at an older age and with a higher comorbidity burden, and large contemporary datasets continue to demonstrate higher rates of adverse early outcomes in women compared to men, with limited evidence of improvement over time (4–7). Importantly, these differences are not only attributable to baseline risk. Registry analyses suggest that women may be less likely to receive guideline-concordant surgical strategies, such as complete revascularization and multi-arterial grafting, which are associated with improved long-term outcomes (6–10). This observation is particularly relevant in the current era, in which the evidence base supporting arterial conduits has strengthened, including individual participant data from randomized trials demonstrating superior long-term clinical outcomes with radial artery (RA) grafting compared with saphenous vein grafting (SVG) (11–13). Together, these findings underscore that sex-related differences in CABG reflect a combination of patient-level risk, procedural strategy, and potentially modifiable care pathways.

In parallel, sternum-sparing minimally invasive coronary surgery has evolved from predominantly single-vessel MIDCAB concepts toward multivessel strategies that aim to maintain complete surgical revascularization while avoiding median sternotomy. Recent case series and comparative studies have supported the feasibility and safety of minithoracotomy-based multivessel CABG, with increasing focus on patient-centered endpoints such as early functional recovery (15, 16).

Total coronary revascularization via left anterior thoracotomy (TCRAT) represents a sternum-sparing, on-pump, arrested-heart multivessel CABG strategy developed to facilitate access to all coronary territories through a single limited thoracic incision, thereby enabling complete anatomical revascularization within a minimally invasive framework. While outcomes of minithoracotomy-based multivessel CABG have been increasingly reported, sex-specific comparative evidence remains limited, and it is currently unclear to what extent sex-related differences observed after conventional sternotomy CABG persist within a standardized sternum-sparing multivessel program.

Therefore, the aim of the present study was to evaluate sex-related differences in baseline characteristics, operative strategy (including conduit selection and extent of revascularization), procedural times, and in-hospital clinical outcomes in a large cohort of patients undergoing minimally invasive multivessel CABG using TCRAT.

## Materials and methods

### Study design and patient population

This retrospective comparative cohort study included consecutive non-emergency patients undergoing minimally invasive multivessel CABG using TCRAT between November 2019 and December 2025. Patients were stratified by sex into a male group (*n* = 807) and a female group (*n* = 138). During the study period, TCRAT was implemented as the standard institutional strategy for eligible patients undergoing isolated multivessel CABG. Conventional sternotomy-based CABG was reserved for emergency cases, redo procedures, or specific anatomical contraindications (extensive calcification of the ascending aorta). The TCRAT procedures were performed by a dedicated team of attending surgeons trained in the standardized technique. There was no sex-related allocation of surgeons, and operative assignment followed routine institutional scheduling.

Given the exploratory nature of this analysis and the relatively low number of adverse events, the statistical analysis was limited to descriptive comparisons between groups. No multivariable adjustment was performed. Accordingly, the analysis was intended as an exploratory comparison of sex-related patterns within the available cohort rather than as a basis for causal inference. To further characterize baseline coronary anatomy, the extent of vessel involvement (2-vessel and 3-vessel disease) was additionally recorded and compared between men and women as part of the descriptive assessment of coronary disease complexity.

### Preoperative evaluation and surgical technique

The preoperative evaluation, anesthesia, and surgical technique of TCRAT have been described by our group previously (14–16); therefore, only key aspects are summarized here.

**Figure 1:**
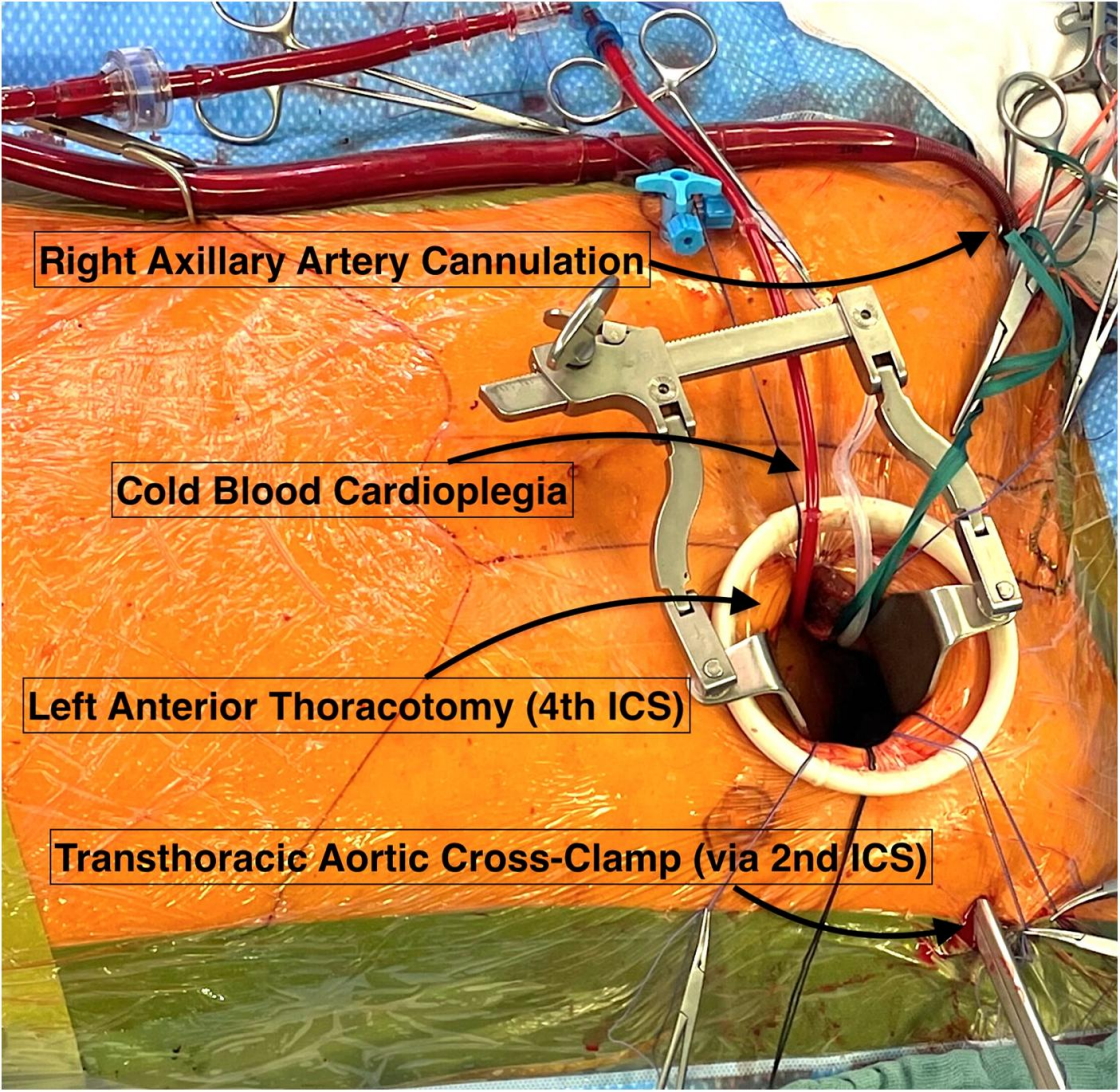
Intraoperative view of on-pump minimally invasive CABG through a left anterior thoracotomy (4th intercostal space) with right axillary arterial cannulation. Myocardial protection is achieved with cold blood cardioplegia, and the ascending aorta is cross-clamped transthoracically through the 2nd intercostal space.

In brief, a preoperative computed tomography angiography was performed in addition to standard institutional examinations to evaluate the ascending aorta, aortic arch, major arterial branches, and iliofemoral vessels. TCRAT consists of three key elements: a sternum-sparing left anterolateral minithoracotomy (typically through the 4th intercostal space); an on-pump approach using a peripheral cannulation strategy enabling stable conditions and cardioplegic arrest; and slinging maneuvers around intrapericardial great vessels to facilitate access to all coronary territories and enable complete anatomical revascularization. When saphenous vein grafts were used, they were generally configured as aorto-coronary conduits with proximal anastomosis to the ascending aorta. When more than one arterial conduit was used, the institutional strategy was based on the left internal thoracic artery and the radial artery as the second arterial conduit. Conduit configuration was determined by the planned extent of revascularization, conduit length, and target-vessel anatomy. In this setting, the radial artery was used either as a free aorto-coronary graft with proximal anastomosis to the ascending aorta or as a composite T-graft. The right internal thoracic artery was not used in the present cohort. The operative setup is shown in Figure 1. In the present cohort, arterial inflow was established uniformly via the axillary artery as part of the standardized institutional TCRAT strategy.

### Definitions and study endpoints

The primary endpoint was in-hospital MACCE, defined as the composite of all-cause mortality, stroke, myocardial infarction, and repeat coronary revascularization during hospitalization.

Secondary endpoints included delirium, atrial fibrillation, pneumonia, bleeding, need for dialysis, ICU length of stay, and hospital length of stay.

### Statistical analysis

Continuous variables are presented as mean (standard deviation) and were compared using Student's *t*-test or Welch's test, as appropriate. Categorical variables are presented as percentages and were compared using *χ*² or Fisher's exact test where appropriate, particularly for rare events.

A two-sided *p*-value < 0.05 was considered statistically significant.

### Ethical standards

This study was approved by the local ethics committee of the University of Marburg (file no. 23–172 RS) and was performed in accordance with the principles of the Declaration of Helsinki (1964) and its later amendments.

All patients provided written informed consent for publication of the study data.

## Results

### Baseline characteristics

A total of 945 patients were included (807 men, 138 women). Women were significantly older (69.2 ± 8.9 vs. 66.7 ± 9.4 years, *p* = 0.004) and had a higher EuroSCORE II (3.9 ± 4.7 vs. 2.7 ± 2.5, *p* = 0.006). Diabetes mellitus was more prevalent in women (44.2% vs. 34.0%, *p* = 0.026). Men were more frequently current smokers (33.2% vs. 21.0%, *p* = 0.004) and had a higher prevalence of prior stroke (4.3% vs. 1.5%, *p* = 0.023). The anatomical extent of coronary artery disease did not differ significantly between sexes. Three-vessel disease was present in 79.4% of men and 77.5% of women (*p* = 0.613), while 2-vessel disease was observed in 20.6% and 22.5%, respectively (*p* = 0.613). Other baseline variables, including BMI and left ventricular ejection fraction, did not differ significantly. Baseline clinical characteristics are presented in Table 1.

**Table 1:** Baseline and clinical parameters.

Variable	Male (*n* = 807)	Female (*n* = 138)	*p*-value
Age, years	66.7 ± 9.4	69.2 ± 8.9	0.004
BMI, kg/m²	28.2 ± 4.3	27.8 ± 5.1	0.404
Ejection fraction, %	49.0 ± 9.5	50.3 ± 8.4	0.078
EuroSCORE II	2.71 ± 2.51	3.85 ± 4.70	0.006
Diabetes mellitus, *n* (%)	274 (34.0%)	61 (44.2%)	0.026
COPD, *n* (%)	133 (16.5%)	17 (12.3%)	0.181
Peripheral arterial disease, *n* (%)	314 (38.9%)	56 (40.6%)	0.711
Prior PCI, *n* (%)	248 (30.7%)	40 (29.0%)	0.684
Current smoker, *n* (%)	268 (33.2%)	29 (21.0%)	0.004
3-vessel disease, *n* (%)	641 (79.4%)	107 (77.5%)	0.613
2-vessel disease, *n* (%)	166 (20.6%)	31 (22.5%)	0.613

Values are mean ± SD or *n* (%). BMI, body mass index; COPD, chronic obstructive pulmonary disease; PCI, percutaneous coronary intervention.

### Operative strategy and procedural times

Sex-related differences were observed in grafting strategy and operative complexity. Men more frequently underwent total arterial grafting (35.8% vs. 21.7%, *p* < 0.001) and received RA as a second arterial conduit (62.2% vs. 35.5%, *p* < 0.001), whereas women received SVG more frequently (78.3% vs. 63.7%, *p* < 0.001). Men also had a higher number of distal anastomoses (3.2 ± 0.8 vs. 2.9 ± 0.9, *p* = 0.002) and more frequent arterial grafting to the left circumflex artery (LCX) and the right coronary artery (RCA) territories (LCX arterial 55.9% vs. 29.7%, *p* < 0.001; RCA arterial 22.8% vs. 10.9%, *p* < 0.001). Additional stratification by the number of distal anastomoses showed a broadly similar distribution of procedural extent between sexes, with numerically more 2-anastomosis procedures in women and more 4-anastomosis procedures in men.

Procedural times were longer in men, including operative time (297.5 ± 79 vs. 277.3 ± 77.7 min, *p* = 0.006), cardiopulmonary bypass (CPB) time (143.1 ± 41.4 vs. 134.8 ± 44.9 min, *p* = 0.032), and aortic cross-clamp time (87.3 ± 32.5 vs. 77.3 ± 32.1 min, *p* < 0.001). Operative characteristics are given in Table 2.

**Table 2:** Operative characteristics.

Variable	Male (*n* = 807)	Female (*n* = 138)	*p*-value
Total arterial grafting, *n* (%)	289 (35.8%)	30 (21.7%)	<0.001
Distal anastomoses, n	3.17 ± 0.84	2.92 ± 0.90	0.002
LAD grafted, *n* (%)	799 (99.0%)	135 (97.8%)	0.362
LAD arterial grafting, *n* (%)	796 (98.6%)	133 (96.4%)	0.172
LCX grafted, *n* (%)	739 (91.6%)	117 (84.8%)	0.036
LCX arterial grafting, *n* (%)	451 (55.9%)	41 (29.7%)	<0.001
RCA grafted, *n* (%)	592 (73.4%)	94 (68.1%)	0.222
RCA arterial grafting, *n* (%)	184 (22.8%)	15 (10.9%)	<0.001
LIMA used, *n* (%)	798 (98.9%)	134 (97.1%)	0.230
Radial artery used, *n* (%)	502 (62.2%)	49 (35.5%)	<0.001
SVG used, *n* (%)	514 (63.7%)	108 (78.3%)	<0.001
2 distal anastomoses, *n* (%)	155 (19.2%)	36 (26.1%)	0.063
3 distal anastomoses, *n* (%)	381 (47.2%)	69 (50.0%)	0.544
4 distal anastomoses, *n* (%)	235 (29.1%)	30 (21.7%)	0.073
5 or more distal anastomoses, *n* (%)	36 (4.5%)	3 (2.2%)	0.255
Operation time, min	297.5 ± 79.0	277.3 ± 77.7	0.006
CPB time, min	143.1 ± 41.4	134.8 ± 44.9	0.032
Cross-clamp time, min	87.3 ± 32.5	77.3 ± 32.1	<0.001

Values are mean ± SD or *n* (%). LAD, left anterior descending; LCX, left circumflex; RCA, right coronary artery; LIMA, left internal mammary artery; CPB, cardiopulmonary bypass.

### In-Hospital outcomes

Most in-hospital outcomes did not differ significantly between sexes. In-hospital mortality was 1.1% in men vs. 0.7% in women (*p* = 0.676). Stroke occurred in 0.7% vs. 1.4% (*p* = 0.404), myocardial infarction in 0.5% vs. 0% (*p* = 0.408), repeat revascularization in 0.5% vs. 1.4% (*p* = 0.366), and MACCE in 1.9% vs. 3.6% (*p* = 0.293). Rates of delirium, atrial fibrillation, pneumonia, bleeding, as well as hospital length of stay did not differ between men and women.

ICU length of stay was longer in men (2.2 ± 4.3 vs. 1.8 ± 1.7 days, *p* = 0.03). Postoperative adverse events and outcomes are presented in Table 3.

**Table 3:** In-hospital outcomes.

Variable	Male (*n* = 807)	Female (*n* = 138)	*p*-value
ICU stay, days	2.2 ± 4.3	1.8 ± 1.7	0.030
Hospital length of stay, days	10.3 ± 8.5	9.7 ± 4.4	0.237
Delirium, *n* (%)	46 (5.7%)	5 (3.6%)	0.248
Atrial fibrillation, *n* (%)	79 (9.8%)	11 (8.0%)	0.512
Pneumonia, *n* (%)	12 (1.5%)	1 (0.7%)	0.478
Bleeding, *n* (%)	48 (6.0%)	8 (5.8%)	0.944
Myocardial infarction, *n* (%)	4 (0.5%)	0 (0.0%)	0.408
Stroke, *n* (%)	6 (0.7%)	2 (1.4%)	0.404
Repeat revascularization, *n* (%)	4 (0.5%)	2 (1.4%)	0.366
In-hospital mortality, *n* (%)	9 (1.1%)	1 (0.7%)	0.676
MACCE, *n* (%)	15 (1.9%)	5 (3.6%)	0.293

Values are mean ± SD or *n* (%). ICU, intensive care unit; MACCE, major adverse cardiac and cerebrovascular events;.(death, stroke, MI, repeat revascularization during index hospitalization).

## Discussion

This analysis examined sex-related differences among patients undergoing sternum-sparing, on-pump minimally invasive multivessel CABG using TCRAT. Three principal findings emerged from this analysis. First, women presented with a higher baseline risk profile, characterized by older age, higher EuroSCORE II, and a higher prevalence of diabetes mellitus. The higher EuroSCORE II observed in women was likely driven primarily by older age and, by definition, by female sex as a component of the score, rather than by broad differences across all recorded baseline comorbidities. Second, clinically meaningful differences were observed in operative strategy: men more frequently underwent a more extensive arterial grafting strategy (higher rate of RA as second arterial conduit and higher rates of total arterial grafting) and received more distal anastomoses, which was accompanied by longer operative, cardiopulmonary bypass, and cross-clamp times. Third, despite these differences in baseline risk and operative strategy, major in-hospital safety endpoints, including mortality, stroke, and MACCE, were comparable between sexes, while ICU length of stay was longer in men. Taken together, these findings suggest that sex-related differences in conduit strategy persist even within a standardized sternum-sparing multivessel CABG program, whereas early postoperative safety appears broadly comparable between women and men. Thus, the principal clinical message of the present study is not that women fare worse with TCRAT, but that a minimally invasive complete revascularization strategy can be applied in women with favorable early results, while important differences in arterial conduit use remain.

Sex-related disparities after conventional CABG have been repeatedly described in large contemporary datasets and meta-analyses, typically showing that women undergo CABG at an older age, with a greater comorbidity burden, and experience higher short-term adverse event rates, including mortality and stroke. Importantly, these differences are not fully explained by baseline risk alone, and recent work has emphasized that variation in care processes and institutional performance may meaningfully modify sex-related outcome gaps (4–7). Despite higher EuroSCORE II and a greater prevalence of diabetes, women in our cohort had in-hospital mortality and stroke rates comparable to men, supporting the feasibility and safety of a standardized on-pump, sternum-sparing TCRAT approach within experienced programs (16).

A key focus of contemporary CABG outcomes research is that sex-related differences are not limited to baseline clinical risk profiles but also encompass variation in operative strategy and surgical quality measures, including arterial conduit application and completeness of revascularization. Analyses from large registries have shown that women are less likely to receive guideline-recommended CABG strategies, including multi-arterial grafting and other practices associated with improved long-term outcomes (6–10). Our data demonstrate a similar pattern within a minimally invasive multivessel CABG setting: women received SVG more frequently, whereas men more commonly received RA grafts as a second arterial conduit and total arterial configurations. These differences are unlikely to be explained solely by the overall extent of coronary artery disease, which was broadly similar between sexes in the present cohort. Rather, they most likely reflect a combination of anatomical and technical considerations together with potentially modifiable aspects of conduit selection in routine surgical practice. Clinically plausible contributing factors include smaller radial artery caliber, conduit-target mismatch, less favorable distal target-vessel quality, diffuse coronary disease, and reduced radial artery availability after prior transradial access. Given the established association between arterial grafting and improved long-term outcomes, more systematic assessment of radial artery suitability and more explicit consideration of arterial grafting in women may help reduce such variation in future practice. These findings are clinically relevant, given the expanding evidence base supporting arterial grafting, particularly RA use, over SVG (11–13). This issue is further underscored by the ongoing ROMA:Women trial, which specifically addresses arterial grafting strategies in female patients undergoing CABG and reflects the growing recognition that sex-specific evidence in this field remains limited. Recent registry and observational data have likewise continued to show that women are less likely to receive multi-arterial grafting despite the potential relevance of arterial conduits for longer-term outcomes (19). Although our analysis is restricted to in-hospital outcomes, conduit choice represents a biologically plausible pathway through which sex-related differences may influence long-term events, graft patency, and the need for repeat revascularization (11–13, 17, 18). Accordingly, the observed conduit-selection gap reinforces the rationale for a dedicated follow-up study addressing midterm and long-term outcomes in this cohort.

The persistence of conduit-selection differences in a sternum-sparing program suggests that surgical approach alone does not eliminate sex-related variation in practice. From a clinical and implementation perspective, this represents a potential quality-improvement opportunity, particularly through more systematic assessment of radial suitability and more structured arterial-grafting strategies in women (8, 19).

Longer procedural times in men likely reflected differences in the extent of revascularization and the greater use of arterial grafting rather than sex itself. Men received more distal anastomoses and more frequent arterial grafting to non-LAD territories, which can plausibly increase operative complexity and duration, particularly in a minimally invasive approach where exposure and sequential grafting strategies are key determinants of time. In the current era, where CABG is increasingly reserved for patients with complex multivessel disease and where arterial grafting is more strongly advocated, procedural duration should be interpreted in the context of the quality and extent of revascularization rather than as an isolated performance metric (1, 2, 9–13). Importantly, comparable major in-hospital adverse event rates suggest that greater operative complexity in men did not result in clinically meaningful differences in early outcomes.

With respect to perioperative morbidity, most secondary endpoints were similar between sexes, while ICU length of stay was longer in men. Contemporary sex-related outcomes literature frequently highlights bleeding/transfusion, stroke, and renal complications as contributors to early disparities; the absence of statistically significant differences in these domains in our cohort is noteworthy but should be interpreted conservatively due to limited power for rare endpoints (4–7). More broadly, as sternum-sparing multivessel CABG programs expand, it is increasingly important that sex-related analyses be integrated into routine outcomes evaluation (20–22).

From a clinical perspective, the present findings highlight that the lower use of arterial conduits in women should not simply be accepted as an inherent feature of treatment. Rather, they point to a need for more deliberate assessment of radial artery suitability and more explicit conduit-selection strategies in routine practice in order to reduce persistent sex-related variation in revascularization strategy.

## Limitations

This study has several limitations. First, it is a retrospective single-center study and may therefore be subject to selection bias. Second, baseline differences between men and women were present, and the analysis was limited to unadjusted comparisons. Third, the relatively small proportion of female patients and the low incidence of adverse events limit the ability to detect small differences between groups. In addition, the marked imbalance in group sizes reduced statistical power for between-group comparisons, particularly for infrequent clinical events, and reinforces that the present analysis should be interpreted as exploratory and descriptive rather than as a basis for causal inference. Patient selection for TCRAT within our program was not sex-specific, and inclusion and exclusion criteria were identical for both groups. Thus, the lower proportion of women in the present cohort most likely reflects the underlying referral and treatment population during the study period rather than intentional procedural selection within the program. However, this distribution may itself reflect broader upstream selection mechanisms operating before entry into the program, including referral patterns, differences in clinical presentation, and preoperative treatment pathway selection, thereby limiting the external validity of the findings. Finally, the analysis was restricted to in-hospital outcomes, and further studies with longer follow-up are warranted to assess the long-term implications of the observed differences in conduit strategy.

## Conclusions

Sternum-sparing multivessel coronary artery bypass grafting via left anterior thoracotomy was associated with similar early postoperative outcomes in men and women despite differences in baseline characteristics and conduit selection. These findings suggest that this minimally invasive revascularization strategy is feasible in both women and men within established minimally invasive CABG programs. However, given the observational design and low event rates, these findings should be interpreted with appropriate caution. Further studies with larger cohorts and longer follow-up are warranted to clarify whether differences in arterial conduit use translate into meaningful variations in long-term outcomes.

## Data Availability

The raw data supporting the conclusions of this article will be made available by the authors, without undue reservation.
